# Wild plants and the food-medicine continuum—an ethnobotanical survey in Chapada Diamantina (Northeastern Brazil)

**DOI:** 10.1186/s13002-021-00463-y

**Published:** 2021-05-26

**Authors:** Patrícia Muniz de Medeiros, Karina Ferreira Figueiredo, Paulo Henrique Santos Gonçalves, Roberta de Almeida Caetano, Élida Monique da Costa Santos, Gabriela Maria Cota dos Santos, Déborah Monteiro Barbosa, Marcelo de Paula, Ana Maria Mapeli

**Affiliations:** 1 Campus de Engenharias e Ciências Agrárias, Universidade Federal de Alagoas, Br 104, s/n, Mata do Rolo, Rio Largo, Alagoas 57100-000 Brazil; 2 Centro das Ciências Biológicas e da Saúde, Universidade Federal do Oeste da Bahia, Rua da Prainha, n 1326, Morada Nobre, Barreiras, Bahia 47810-047 Brazil; 3 Centro de Biociências, Universidade Federal de Pernambuco, Av. Reitor Joaquim Amazonas, s/n, Cidade Universitária, Recife, Pernambuco 50740-570 Brazil; 4 Instituto de Ciências Biológicas e da Saúde, Universidade Federal de Alagoas, Av. Paulo Holanda, 143, Cidade Universitária, Maceió, Alagoas 57072-900 Brazil; 5 Centro das Ciências Exatas e das Tecnologias, Universidade Federal do Oeste da Bahia, Rua da Prainha, n 1326, Morada Nobre, Barreiras, Bahia 47810-047 Brazil

**Keywords:** Ethnobotany, Traditional knowledge, Functional foods, Wild edible plants, Selection criteria

## Abstract

**Background:**

Ethnobotanical research has demonstrated that several wild food plants (WFP) are used for medicinal purposes. Therefore, in addition to constituting an important source of nutrients, WFP can be used to help treat and avoid health problems. This study sought to characterize the traditional use of plants considered simultaneously as food and medicine by local specialists in the community of Caeté-Açu, which borders Chapada Diamantina National Park (NE Brazil). We also sought to identify the variables that influence the species’ cultural importance.

**Methods:**

We selected local specialists based on a snowball sample and used a free-listing technique to register the wild plants they knew that are both edible and medicinal. Then, we asked the specialists to rank each plant component cited according to the following attributes: (1) ease of acquisition, (2) taste, (3) smell, (4) nutritional value, and (5) medicinal value. We used multiple regression to determine the variables that influence the cultural salience.

**Results:**

The most culturally salient species was *Anredera cordifolia* (Ten.) Steenis*.* The main medicinal effects associated with this species were related to body strengthening, intestinal regulation, and stomach issues. The most salient used species were those that were easiest to acquire and had the highest perceived nutritional values.

**Conclusion:**

It is likely that the sociocultural backgrounds of the respondents (elders, former miners, or descendants of miners) and the historical importance of wild food plants to local diets increased the predictive power of the perceived nutritional importance and ease of acquisition of these plants.

**Supplementary Information:**

The online version contains supplementary material available at 10.1186/s13002-021-00463-y.

## Background

In many cases, medicinal and food uses of natural resources are so interconnected that is difficult to establish when one use ends and the other begins [1]. Ethnobotanical studies have addressed this food-medicine continuum by evaluating the overlap between medicinal and edible uses in several social-ecological systems [2–5]. Although a large number of species are exclusively medicinal or exclusively edible, investigations have discovered outstanding intersections, which have been sufficient to relativize previous ideas of the separation between them.

Different forms of interactions between food and medicine may emerge in social-ecological systems. Pieroni and Quave [6], in a study including Albanians and Italians in Lucania (Southern Italy), synthesized such relations in three possible contexts: (1) a given plant is used both as medicine and food but without any further association between the two (e.g., the plant component used for medicinal purposes is different from the part consumed as food); (2) a food plant is considered healthy but without a unique or specific medicinal target (a so-called “functional food” according to some definitions); and (3) a plant is consumed to obtain a specific medicinal effect (a medicinal food or food medicine).

Although several ethnobotanical studies have directed efforts to understand this food-medicine continuum, there is a lack of investigations dedicated to finding the main variables related to the species’ cultural importance^1^ in such a context. Most studies are focused on eliciting the reasons behind the maintenance and abandonment of the traditional uses of these products as a whole and do not focus on specific differences in cultural importance among plants. Such studies have indicated sociocultural aspects, health claims, and availability as important factors [7–9].

Regarding the differences in cultural importance among species, research on food plants (in general or focused on specific groups like fruits) has elucidated the relevance of several influential variables, such as taste [10, 11], availability in time or space/ease of acquisition [11, 12], nutritional value [11, 13], easiness to process [13], and associated uses—including medicinal uses [11, 12]. Studies on medicinal plants have also indicated the role of their organoleptic properties [14–16] and availability [16, 17] as well as the importance of their therapeutic efficiency or the presence of certain compounds [16, 18, 19].

Most studies on medicinal and food uses access this information by directly asking respondents the reasons why they consume (or have abandoned the consumption of) certain species [10, 20]. This approach has provided evidence of important aspects of interactions between people and plants and is a powerful tool in the elucidation of cultural and context-specific drivers of changes in species’ cultural importance.

An alternative approach uses statistical tools (predictive models) to find a possible causal relation between one (or several) explanatory variable(s) and a response variable. In such cases, the response variable is often an indicator of the species’ cultural importance (knowledge or use), and the explanatory variables are attributes that can be measured with ecological tools (e.g., availability as measured by the species’ relative density, using data from plant inventories) or chemical tools (e.g., therapeutic efficiency as measured by the presence of certain bioactive compounds) or are based on people’s perception [11, 16–18]. While this approach neglects the direct opinions of respondents, it has the advantage of capturing variables that may operate unconsciously or even variables that are culturally rooted but have environmental or biological meaning (e.g., when people mention that some plants are more important because of tradition, but those plants are those with higher energy content). It means that when people are asked about the reasons why some species are more culturally important than others, they may omit factors that do not operate consciously. However, when we use a statistic model to find relationships between variables, such unconscious or culturally rooted motivations may be elicited.

Species availability strongly influences plant knowledge and use for different purposes, especially woody uses [21], but has been a weak predictor of the cultural importance of plants for medicinal purposes [21] and an even weaker predictor of food uses [22, 23]. However, for both medicinal and food purposes, recent studies using the local perception of availability instead of conventional ecological tools have found that availability may not be disregarded in regard to predicting species’ relevance for medicinal and food purposes [11, 16].

In many cases, people usually attribute greater importance to organoleptic characteristics (particularly taste) than other characteristics when choosing which food plants to consume [8, 24, 25]. Taste and smell may also provide cues for identifying the therapeutic potential of plant species, which is an important influencer of the use of plants for medicinal purposes [26, 27]. Taste also plays an important role in indicating the medicinal properties of a given food. For example, some studies have found that bitter food medicines are often consumed to treat diabetes as the bitter taste counterbalances the sweetness of the disease [28, 29].

Studies have also displayed great variation in terms of the main variables associated to the plant cultural importance. Therefore, we need to increase case studies focusing on searching for such variables to better understand situations in which each one gains or loses predictive power over the species’ cultural importance. Considering the specific group of plants for which medicinal and food uses overlap, much work remains to be performed to discover whether therapeutic or nutritional value is primary when deciding which plants to consume.

For this reason, we aimed to characterize the traditional use of wild plants that are both food and medicine by local specialists in a rural community bordered by the Chapada Diamantina National Park (NE Brazil). We also chose four criteria described in the scientific literature (smell, taste, nutritional importance, and medicinal importance) to determine possible variables associated to the cultural salience of such species.

The relationship between plant cultural importance and the species characteristics may not be considered as a one-way process. Literature has shown that people tend to favor the abundance and distribution of useful plant species [30]. Additionally, people’s management over time has led to genetic changes, altering plant characteristics (morphology, physiology, phenology, life cycles) according to different human requirements [31]. Examples can include the alterations in the amount of certain compounds [32, 33], which can, in turn, have an influence on a plant’s taste, nutritional/medicinal use, and toxicity.

Considering that a plant’s cultural importance may act as both cause and consequence of its characteristics, our approach is clearly a cut-off of the whole set of processes that involves the above-cited variables and the species’ cultural importance. Moreover, given that people promote in situ plant management in natural areas, the concepts of wild and domesticated may not be considered as discrete entities. Literature has indicated a continuum between the wild and domesticated conditions, with several levels of management intensity between them [31, 34, 35]. For this reason, many species gathered in natural areas may not be considered as completely wild. We adopted the emic perspective of wild plants, which led to the inclusion of species that were locally classified as wild, as performed by previous studies [34]. This approach may have led to the inclusion, for example, of semi-domesticated species, but this formal classification was not among the aims of our study.

## Methods

### Study area

**Fig. 1:**
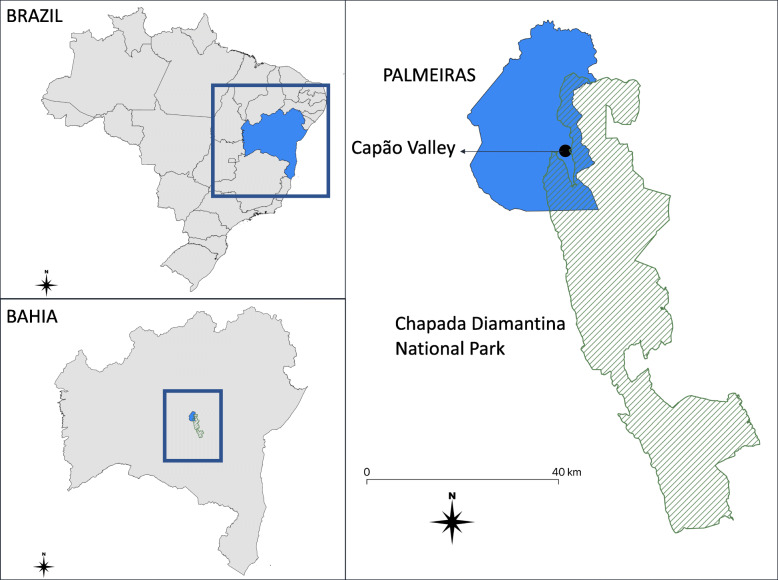
Location of the Capão Valley, adjacent to Chapada Diamantina National Park, south-central Bahia State, Northeastern Brazil

We conducted this study in the rural community of Caeté-Açu (also known as the Capão Valley). The valley is located in the Palmeiras municipality (south-central Bahia State, Northeastern Brazil) (Fig. 1). It is situated in the geographical region of Chapada Diamantina (“Plateau of Diamonds”) and surrounds Chapada Diamantina National Park.

This region has a rugged relief; its altitude varies from 200 to 2033 m above sea level, and it is 500 m above sea level (Franca Rocha et al. 2005). In most of Chapada Diamantina, the climate is humid tropical with dry winters and temperate summers (Cwb climate according Köopen’s classification) [36]. Most of the annual rainfall is concentrated in summer.

The ecosystems of Chapada Diamantina form a mosaic of different vegetation types, such as c*ampos rupestres*, submontane to montane forests (including riparian forests), Cerrado (a Brazilian savanna), Caatinga, and wetlands. *Campo rupestre* is open vegetation composed of sclerophyllous and evergreen shrubs or subshrubs and small trees, situated at the highest altitudes [37]. The submontane to montane forests include vegetation with high continuous canopies (ranging from 8.5 to 20 m high), a lower canopy with shrubs and small trees, and an open understory with rare epiphytes [38]. The Cerrado is characterized by a discontinuous arboreal layer up to 10 m tall, with twisted branches and thick tree bark. The ground layer is usually continuous, composed mainly of grasses, sedges and numerous subshrubs [37]. Caatinga vegetation is highly xeromorphic with a predominance of profusely branched low trees and shrubs. Most of the plants in the Caatinga areas have small leaves, spines, thick bark, and a well-developed root system often bearing tubers [37]. The wetlands are permanently or periodically inundated or boggy areas [37].

Habitat heterogeneity and anthropogenic disturbances are factors that generate high floristic heterogeneity in the local ecosystems [39, 40]. Therefore, there is high beta diversity in the region (change in community composition along environmental gradients).

### Sociocultural setting

Before the Portuguese colonization (century XVI), the Chapada Diamantina region (which at that time belonged to a region named *Sertão das Jacobinas*) was inhabited by different ethnic groups, such as the Payayá, Sapoiá, Tocó, Moritises, Maracás, Secaquerinhens, Cacherinhens, Caimbés, Pankararu, Ocren, Oris, Tamaquins, Araquenas, Anaiós, and Topins [41]. As in other hinterland northeastern regions, the Portuguese colonization of the *Sertão das Jacobinas* was positively impacted by the cattle ranching (Santos, 2011). In the middle of the XIX century, the discovery of diamonds in the region spurred a huge migration wave (including Europeans, enslaved Africans, and Brazilians from many regions, mainly from the southeast and northeast) and the establishment of large villages in Chapada Diamantina [42].

Mining began to decrease in the area near the end of the XIX century, and many people migrated to Southern and Southeastern Brazil to work in coffee cultivation (Matta, 2006). However, local livelihoods were so strongly linked to the mining activity that some people migrated to the state of Mato Grosso do Sul searching for other diamond mines, while others continued to mine in Chapada Diamantina against all odds [43].

In the 1980s, Capão began to be a common site for ecotourism [44]. Then, strong migration took place in the valley, mainly by people seeking to live in contact with nature and who were looking for a healthy lifestyle [44]. The valley is currently inhabited by people from several countries (in particular, from Latin America and Europe) and provinces [44]. These people coexist with native people, mostly descendants of miners [44]. Native residents are a racially mixed group of descendants from African, European, and indigenous peoples.

Natives and migrants currently work in tourism (as guides, or in related services—restaurants, bars, hotels, etc.), small-scale agriculture, and other services and commercial enterprises. Among the elders, many of the native residents are former miners who continued to mine even after the decrease in its productivity and that are currently retired. The shift in the village’s economic activities and the creation of the Chapada Diamantina National Park in 1985 led to changes in the way people interact with the native vegetation areas, causing a decrease in the number of people who harvest plant resources from these areas, and in the frequency of harvesting events.

According to the local health center, there were 693 families and 1177 people living in the community of the Capão Valley in 2014. The main economic activities of these people are related to commerce and tourism, but small-scale agriculture is still common. People can access official medical facilities in downtown Palmeiras (21 km from Capão) and in neighboring cities (Seabra, Lençóis, and Iraquara) [45]. A great diversity of religions and beliefs is found in Capão. Catholicism and Protestantism are predominant, although eastern religions (e.g., Buddhism and Shintoism) and Santo Daime are increasing [45].

### Data collection

We conducted data collection between April and June 2014. We used the snowball technique to select local specialists. We performed informal interviews that led to the recognition of a first local expert^2^, which was acknowledged by several people in the community. This expert indicated another one and so on until there was no other specialist mentioned (*N* = 13, nine men and four women). Therefore, our results may not be interpreted as a pattern for the whole community but rather as a tendency for local experts. This technique led to the inclusion of older individuals (from 56 to 84 years old). All the interviewees were born in the Chapada Diamantina region. The interviewees either did not have formal education or attended only basic school. Among the male interviewees, most are currently retired from mining and farming activities. Female respondents were housewives and/or retired from agricultural activities. There was only one exception from the dominant (past or present) mining/farming/housekeeping activities, given that one male respondent worked as both farmer and mechanic. All the interviewees were descendants of former miners.

We explained to the key informants that we were interested in understanding their knowledge and opinions about the wild edible plants they knew that also had health benefits. Then, we used the free-listing technique to stimulate the informants to list the plants they knew and the plant parts they used. In some cases, the respondents cited more than one plant part for a single species, which is why we employed the “plant-part” pairs for the other steps of our interview. For example, the species *Piper umbellatum* L. had both leaves and roots as the edible parts. Therefore, we considered the pair *P. umbelatum*-leaf as a different entity from the pair *P. umbeltum*-root.

The respondents indicated the medicinal benefit of each of the cited plant-part pairs. Then, we asked the interviewees to rank the plant-part pairs they cited according to the following criteria (one at a time): (1) ease of acquisition, (2) taste, (3) smell, (4) nutritional value, and (5) medicinal value. All of these criteria were evaluated based on the local experts’ perceptions. As we dealt with ranks, the interviewees attributed a value of 1 to the best species for a given attribute (e.g., the most frequently consumed, the most tasteful, etc.) and subsequent values to other species until the “worst” plant for a given attribute was assigned a rank n (n=total length of the respondent’s free list). We also permitted ties (e.g., when a respondent indicated the first and second most nutritive plant-part pairs and stated that the other four all had equal nutritive value, we considered the ranks as 1, 2, 3, 3, 3, 3). The rankings of all 13 respondents are available in Supplementary file 1.

We adapted our questions to the local context to avoid misinterpretations. For example, instead of asking respondents to rank species according to their nutritional importance, we asked them to rank plants from those that provided more “sustenance” (*sustância*) to those that provided less sustenance. Additionally, instead of asking for the species’ medicinal importance, we asked the interviewees to rank species from those that were better medicines to those that were worst medicines, regardless of the therapeutic indication. We chose the best emic terms based on our previous experience in the research area and informal interviews prior to our free lists.

As we used the term *sustância*, we were not able to capture the full perceived nutritional importance of the wild food plants. The term is related to the foods that are popularly considered strong and energetic [46, 47], sustaining people for longer periods after ingestion and avoiding hunger [47]. Therefore, by using this term, we are capturing perceptions about satiety instead of the presence of certain nutrients. We used satiety as a proxy to the nutritional importance in its energetic dimension.

We used “ethnospecies^3^” as our research unit. Only the top 5 culturally salient species were identified. Vouchers were deposited in the Dárdano de Andrade Lima Herbarium (Empresa Pernambucana de Pesquisa Agropecuária) and in the Professor Vasconcelos Sobrinho Herbarium (Universidade Federal Rural de Pernambuco).

### Data analysis

Before statistical analysis, we excluded plant-part pairs cited by fewer than three local experts to avoid biases from idiosyncratic information, leaving twenty-one plant-part pairs. We summarized each of the aforementioned attributes according to the mean value (the sum of the ranks attributed to the plant-parts divided by the number of interviewees that mentioned the plant). Given that a respondent could not provide information about a species he/she did not mention, the number of respondents used to calculate the mean values for an ethnospecies was exactly the number of individuals who mentioned it.

The cultural salience was our indicator of cultural importance. Salience was calculated according to Smith and Borgatti [49]. The index takes into account both the frequency and the rank order of plants in the lists. Therefore, the most salient species were those that (1) were cited by several people and (2) were among the first plants to be recalled by the interviewees. The index has been used by previous ethnobiological studies as an indicator of the cultural importance of plant species (see, for example, Ghorbani et al. [50]).

We used a multiple regression model to assess the variables that influence cultural salience. Multiple regression is a statistical procedure employed to analyze the relationship between a single dependent variable (in this case—cultural salience) and several independent variables (ease of acquisition, taste, smell, and nutritional and medicinal importance).

We used a stepwise approach to include in each final model only the variables that provided the lowest AIC^4^ (Akaike information criterion) value for the model. We used the “vif” function in the R package “car” to detect multicollinearity. This function returned the Variance Inflation Factor (VIF) for our explanatory variables, and all the values were <5, indicating the absence of strong multicollinearity.

We standardized the final model by centering and scaling the data. This procedure allowed for better comparisons among the coefficients.

## Results

We identified 57 plant-part pairs belonging to 46 ethnospecies in the intersection between medicinal and food plants in the region. From these, 21 plant-part pairs from 20 ethnospecies were cited by three or more respondents (Supplementary file 1) .

Regarding the plant parts used, the respondents mentioned fruits for 47.8% of the ethnospecies, followed by leaves (39.1%), roots (21.7%), and other parts (15.2%). Most of these foods are consumed *in natura*, as juices, in salads, as sweets or as condiments. Respondents considered a wide range of health benefits of wild foods. The medicinal indication associated with the highest number of ethnospecies was body strengthening (52.2% of the ethnospecies), followed by intestinal regulation (37%), treatment of stomach issues (26.1%), improvement of kidney function (13.0%), and calming properties (10.9%). The position of body strengthening as the health benefit associated with the most species indicates that functional foods are highly valued among local experts. However, among the top 5 most salient species, the main medicinal indications were target-specific (intestinal regulation and calming properties), which shows that to achieve high cultural importance in the food-medicine system a specific medicinal effect is often required.

The species with the highest value for cultural salience was *Anredera cordifolia* (Ten.) Steenis, followed by *Passiflora edulis* Sims, *Ipomoea serrana* Sim-Bianch. & L.V.Vasconcelos, *Passiflora cincinnata* Mast., and *Piper umbellatum* L (Table 1). Interestingly, four of the top 5 species are vines.

**Table 1:** Functional and medicinal foods with the highest values for cultural salience as mentioned by local experts in the rural community of Caeté-Açu. NE Brazil. Med, main medicinal target

TOP-5 salience	Family	Popular name	Life form	Used part	Salience	Med	Voucher n°
*Anredera cordifolia* (Ten.) Steenis	Basellaceae	Quiabinho	Vine	Leaf	0.48	Intestine	^a^
*Passiflora edulis* Sims	Passifloraceae	Maracujina	Vine	Fruit	0.48	Calming	IPA 90073
*Ipomoea serrana* Sim-Bianch. & L.V.Vasconcelos	Convolvulaceae	Batata da serra	Vine	Root	0.43	Intestine	^a^
*Passiflora cincinnata* Mast.	Passifloraceae	Maracujá do mato	Vine	Fruit	0.39	Calming	IPA 90036
*Piper umbellatum* L.	Piperaceae	Capeba	Sub-shrub	Leaf	0.37	Liver	IPA 90188

^a^Not deposited (unfertile material)

The stepwise approach left the variables nutritional value and ease of acquisition in the final model. The model explained an intermediate amount of variation in the response variable (R^2^=0.47; Adjusted R^2^=0.41), and the AIC value was low (−36.2). Both variables exerted a significant influence on cultural salience, but ease of acquisition exhibited a slightly higher effect (Table 2).

**Table 2:** Predictors of the cultural salience of functional and medicinal foods known by local experts in the rural community of Caté-Açu, Chapada Diamantina, NE Brazil

Variable	Estimate	S.E.	t value	***P***
Intercept	0.26	0.02	13.04	0.00
Mean ranking for ease of acquisition	– 0.06	0.02	– 2.58	0.02
Mean ranking for nutritional value	– 0.05	0.02	– 2.18	0.04
AIC	– 36.2			
R^2^	0.47			
Adjusted R^2^	0.41			

For the two variables, there was an inverse relationship, meaning that lower values for the mean ranks led to higher salience values. Lower values (close to one) of the mean ranks indicated that species were often cited in the first positions. Therefore, a higher perceived nutritive value and ease of acquisition were positively associated with cultural salience.

## Discussion

This study strengthens the idea that food and medicinal plants constitute a continuum, given that it indicated a large number of plants involved in this intersection. However, in terms of use patterns, the plants within the continuum are more closely related to the food domain that the medicine domain. The status of fruit as the major plant part follows a pattern common for wild food plants in the tropics [51–53] and in other areas. Moerman [3], for example, in a study on food and medicinal plants used by the Native North Americans, found that “Fifty-eight percent of food uses are of fruits, while only 8% of medical uses are of fruits.” Such results provide evidence that different plant parts can be used for different purposes in the same region. For medicinal plants in Chapada Diamantina, the most common pattern is the outstanding presence of leaves and barks, with a low representation of fruits [54, 55].

Our findings suggest that ease of acquisition and nutritional importance are the most important determinants of the knowledge and consumption of wild functional and medicinal foods by local experts from the community of Caeté-Açu. Ease of acquisition is important not only in terms of specific differences in plant cultural importance but also in terms of explaining the abandonment of a whole set of wild foods and medicines [8], as in many regions deforestation is distancing people from natural resource areas.

Studies using ecological indicators of availability to test their relationships with species’ cultural importance have not indicated ease of acquisition as an important predictor [22, 23]. Our study strengthens the idea that accessing people’s perceptions of availability instead of ecological data may provide evidence of certain patterns not highlighted by the latter technique, which may happen because incorporating perceptions of local people allows for a wider number of plants in the analyses, given that wild species occurring outside of forest areas may also be included, as well as species with different life forms. Studies including ecological data commonly focus on specific life forms—more often on woody plants [22, 23]. Furthermore, high values of ecological abundance or frequency do not necessarily ensure that the plants are easy to acquire since they can occur in areas difficult to access. At the same time, low values of ecological abundance or frequency may not indicate that the species is difficult to acquire, as it may occur precisely on people’s common pathways.

However, such arguments do not intend to infer that local perception is a better indicator of species availability/ease of acquisition than ecological indices. Under some circumstances, studies have found correlations between the cultural importance of plant species and ecological indices, while they have not found a significant correlation between the plants’ cultural importance and the local perception of availability. In a case study on the factors that influence fuelwood use, Hora et al. [56] encountered this situation and inferred that most of their interviewees were older people who were no longer accessing forest areas and acquired fuelwood from younger community members. Therefore, their perceptions of availability could be strongly related to a past landscape, which could weaken the relationship between perceived availability and fuelwood use. Therefore, we need to perform more studies to understand which portions of availability each technique (ecological indicators *vs* local perception) is specifically capturing.

Nutritional value has proven to be an important variable that influence wild food plant cultural importance in other contexts [11, 13]. The fact that nutritional value remained in the final model, while medicinal value did not, shows that, although local experts recognize the important of medicinal attributes in wild food plants, nutritional aspects are more relevant for designating culturally important plants in the food-medicine continuum than medicinal properties.

Our results are consistent with the social-ecological theory of maximization. One of the theory’s assumptions is that the most important natural resources to local people are those that afford the maximum return, considering the balance among different explanatory variables that affect resource use [57]. This maximum return is the best possible achievement from the combination of different variables driving the use of a natural resource [57]. Additionally, the most important variable as evidenced by our research are also among those deeply studied in the context of optimal foraging theory [58]—in which nutritional value is analogous to energy intake and ease of acquisition is analogous to energy spent during foraging. Therefore, a balance between the costs of acquiring a plant and the nutritional benefits of consuming it seems to influence cultural relevance in the intersection between food and medicine.

Contrary to other studies, attributes such as taste did not explain plants’ cultural importance. The higher importance of nutritional and availability issues compared to taste may be explained by the sociocultural background of the community. Most of the elders (including those from our sample) are former miners or descendants of former miners. In the region, miners worked for extended periods, forcing them to spend much time in natural areas, which were often far from settlements. Wild plants therefore constituted an important source of food in the mining context with a better return for miners implicit in the consumption of nutritionally rich, widely available species. This process probably influenced the importance of plant species, which was transmitted culturally to the miners’ descendants. For this reason, nutritional value and ease of acquisition remain important factors that influence plant cultural importance even now when these inhabitants do not rely much on wild foods as an important source of daily energy intake.

However, we need to conduct further studies including nonspecialists (particularly the younger members of the community) to provide evidence for the main factors that influence plants’ cultural importance in the intersection between food and medicine. It is possible that the culturally mediated effects of nutritional value and ease of acquisition lose strength among younger dwellers, who are mostly unfamiliar with mining activity and never consumed wild plants as an important source of energy intake. Among people who consume wild plants as a supplementary source of food, taste may be more important, considering that their energy intake is probably being fulfilled by other (nonwild) foods. However, this claim needs to be tested using proper research designs.

Several other variables that were not included in our research may be associated to the plants’ cultural importance in this food-medicine system. Moreover, qualitative aspects that we did not access can affect the use of food items. People have emotional links (both positive and negative) to food related to childhood memories, family togetherness, sharing care through foods, amusing cooking times or mealtimes, anxiety, or sadness [59]. These memories may act as bridges between past individual experiences and present life, affecting the way an individual approaches food [59]. For some individuals from rural communities, eating wild foods can elicit positive memories related to early childhood or can cause them to feel more connected to nature [34, 60]. In such cases, certain food/medicine items may be culturally important because people can experience strong emotional ties to them. Such aspects may not be properly captured by our research design, which is definitely a limitation of our study.

This study has some other limitations. The investigation would have been a stronger tool for finding variables that influence plant use if the conventional approach (of directly asking people their reasons for preferring/consuming certain items) had been combined with our statistical model. Therefore, we strongly recommend this combination of approaches for future studies on this subject. Additionally, our sample of ethnospecies was small, which makes our model with two independent variables not as reliable as it would have been if it had included a high number of plants. Finally, other options to classify plants by the attributes would have been preferable over ranking. We obtained a high number of ties (i.e., people stating that several species occupied the same position in terms of taste, for example). When ties are common, scoring exercises [11] are much better options than rankings.

## Final remarks

Our findings demonstrate that for local experts in Chapada Diamantina, the cultural importance of particular plants in the intersection between food and medicine follows a logic of maximum return, so that species with better returns in terms of ease of acquisition and nutritional value have been culturally appropriated and rooted. Furthermore, even considering the intersection between food and medicine, the evaluation of use patterns (e.g., the major use of fruits) and the variables that influence plant’s cultural importance (e.g., nutritional instead of medicinal importance) indicate that this group of species is more culturally associated with the domain of food than the domain of medicine. Finally, additional studies are needed to accurately place the food-medicine intersection within social-ecological systems.

## Supplementary information

**Additional file 1.** Ranking_Taste.

## Data Availability

Raw data is available in the Supplementary file 1.

## Abbreviations

WFP: Wild food plants
NE: Northeast
AIC: Akaike information criterion

## Footnotes

1 A plant’s cultural importance has been measured with several different approaches, that often leads to correlated results (see, for example, Tardío and Pardo de Santayana [7], but may also strongly differ. When we use this term, we are considering this whole set of approaches and indices, but our study specifically used cultural salience as a way to access cultural importance.

2 We considered as a local expert a person that was perceived by community members as a possessor of a vast knowledge in the domains of medicinal and food plants.

3 We considered ethnospecies as biological entities recognized and designated by a human group, not necessarily corresponding to a biological species from a taxonomic perspective [48].

4 The AIC “measures the distance, so to speak, between a candidate model and the true model—the closer the distance, the more similar the candidate to the truth”. The stepwise approach used in this study identifies the best model based on the AIC, i.e., the variables that together provide the best fit (lower AIC) to the model. In the case of our study, we tested the influence of five variables over the cultural importance of plant species. The best model retained only two variables, meaning that the remaining three are probably not as important predictors of the cultural importance as the variables that were kept in the model.

## References

[1] Pieroni A, Price LL, Pieroni A, Price LL (2006). Introduction. Eat Heal Tradit Food As Med.

[2] Etkin NL, Ross PJ (1982). Food as medicine and medicine as food. An adaptive framework for the interpretation of plant utilization among the Hausa of northern Nigeria. Soc Sci Med..

[3] Moerman DE (1996). An analysis of the food plants and drug plants of native North America. J Ethnopharmacol..

[4] Pieroni A, Quave CL (2005). Traditional pharmacopoeias and medicines among Albanians and Italians in southern Italy: a comparison. J Ethnopharmacol..

[5] Pieroni A, Sheikh Q-Z, Ali W, Torry B (2008). Traditional medicines used by Pakistani migrants from Mirpur living in Bradford, Northern England. Complement Ther Med.

[6] Pieroni A, Quave CL, Pieroni A, Price LL (2006). Functional foods or food medicines? On the consumption of wild plants among Albanians and Southern Italians in Lucania. Eat Heal Tradit Food As Med.

[7] Tardio J, Pardo-de-Santayana M (2008). Cultural importance indices: a comparative analysis based on the useful wild plants of southern Cantabria (northern Spain). Econ Bot..

[8] Sõukand R (2016). Perceived reasons for changes in the use of wild food plants in Saaremaa, Estonia. Appetite.

[9] Towns AM, van Andel T (2016). Wild plants, pregnancy, and the food-medicine continuum in the southern regions of Ghana and Benin. J Ethnopharmacol..

[10] Gi S, Calvet-Mir L, Carrió E, D’Ambrosio U, Garnatje T, Parada M (2016). A matter of taste: local explanations for the consumption of wild food plants in the Catalan Pyrenees and the Balearic Islands. Econ Bot..

[11] Gomes DL, Santos Ferreira RP, Santos EMC, Silva RR, Medeiros PM (2020). Local criteria for the selection of wild food plants for consumption and sale in Alagoas, Brazil. Ethnobiol Conserv.

[12] Campos L, Nascimento A, Albuquerque U, Araujo E (2016). Criteria for native food plant collection in Northeastern Brazil. Hum Ecol..

[13] Balemie K, Kebebew F (2014). Ethnobotanical study of wild edible plants in Derashe and Kucha Districts, South Ethiopia. J Ethnobiol Ethnomed.

[14] Ankli A, Sticher O, Heinrich M (1999). Medical ethnobotany of the Yucatec Maya: healers’ consensus as a quantitative criterion. Econ Bot..

[15] Molares S, Ladio A (2014). Medicinal plants in the cultural landscape of a Mapuche-Tehuelche community in arid Argentine Patagonia: an eco-sensorial approach. J Ethnobiol Ethnomed..

[16] Caetano RA, Albuquerque UP, Medeiros PM (2020). What are the drivers of popularity and versatility of medicinal plants in local medical systems?. Acta Bot Brasilica..

[17] Lucena RFP, Araújo EL, Albuquerque UP (2007). Does the local availability of woody caatinga plants (Northeastern Brazil) explain their use value?. Econ Bot..

[18] Monteiro JM, de Souza JSNJSN, Neto EMFL, Scopel K, Trindade EF, Lins Neto EMF (2014). Does total tannin content explain the use value of spontaneous medicinal plants from the Brazilian semi-arid region?. Rev Bras Farmacogn..

[19] Omar S, Lemonnier B, Jones N, Ficker C, Smith ML, Neema C (2000). Antimicrobial activity of extracts of eastern North American hardwood trees and relation to traditional medicine. J Ethnopharmacol..

[20] Leal ML, Alves RP, Hanazaki N (2018). Knowledge, use, and disuse of unconventional food plants. J Ethnobiol Ethnomed.

[21] Gonçalves PHS, Albuquerque UP, Medeiros PM (2016). The most commonly available woody plant species are the most useful for human populations: a meta-analysis. Ecol Appl..

[22] Thomas E, Vandebroek I, Van Damme P (2009). Valuation of forests and plant species in indigenous territory and National Park Isiboro-Sécure. Bolivia. Econ Bot..

[23] Phillips O, Gentry AH (1993). The useful plants of Tambopata, Peru: II. Additional hypothesis testing in quantitative ethnobotany. Econ Bot..

[24] Ghirardini MP, Carli M, Vecchio N, Rovati A, Cova O, Valigi F (2007). The importance of a taste. A comparative study on wild food plant consumption in twenty-one local communities in Italy. J Ethnobiol Ethnomed.

[25] Thakur D, Sharma A, Uniyal SK (2017). Why they eat, what they eat: patterns of wild edible plants consumption in a tribal area of Western Himalaya. J Ethnobiol Ethnomed.

[26] Ankli A, Sticher O, Heinrich M (1999). Yucatec Maya medicinal plants versus nonmedicinal plants: indigenous characterization and selection. Hum Ecol..

[27] Brett JA, Heinrich M (1998). Culture, Perception and the environment: the role of chemosensory perception. J Appl Bot..

[28] Jennings HM, Merrell J, Thompson JL, Heinrich M (2015). Food or medicine? The food–medicine interface in households in Sylhet. J Ethnopharmacol..

[29] Pieroni A, Sheikh Q, Ali W, Torry B. Traditional medicines used by Pakistani migrants from Mirpur living in Bradford, Northern England. Complement Ther Med. 2008;16:81–6. 10.1016/j.ctim.2007.03.005 18514909

[30] Levis C, Flores BM, Moreira PA, Luize BG, Alves RP, Franco-Moraes J, et al. How people domesticated Amazonian forests. Front Ecol Evol. 2018;5:171.

[31] Casas A, Del Carmen VM, Viveros JL, Caballero J (1996). Plant management among the nahua and the mixtec in the Balsas River Basin, Mexico: an ethnobotanical approach to the study of plant domestication. Hum Ecol..

[32] Bradbury EJ, Emshwiller E (2011). The role of organic acids in the domestication of oxalis tuberosa: a new model for studying domestication resulting in opposing crop phenotypes. Econ Bot.

[33] Urbina CJF, Casas A, Martínez-Díaz Y, Santos-Zea L, Gutiérrez-Uribe JA (2018). Domestication and saponins contents in a gradient of management intensity of agaves: Agave cupreata, A. inaequidens and A. hookeri in central Mexico. Genet Resour Crop Evol..

[34] Cruz-Garcia GS, Price LL (2014). Human-induced movement of wild food plant biodiversity across farming systems is essential to ensure their availability. J Ethnobiol..

[35] González-Insuasti MS, Caballero J (2007). Managing plant resources: how intensive can it be?. Hum Ecol..

[36] Alvares CA, Stape JL, Sentelhas PC, Moraes Gonçalves JL, Sparovek G (2013). Köppen’s climate classification map for Brazil. Meteorol Zeitschrift..

[37] Funch RR, Harley RM, Funch LS (2009). Mapping and evaluation of the state of conservation of the vegetation in and surrounding the Chapada Diamantina National Park, NE Brazil. Biota Neotrop.

[38] Funch LS, Funch R, Barroso GM (2002). Phenology of gallery and Montane Forest in the Chapada Diamantina, Bahia, Brazil1. Biotropica..

[39] Couto APL, Funch LS, Conceição AA (2011). Composição florística e fisionomia de floresta estacional semidecídua submontana na Chapada Diamantina, Bahia, Brasil. Rodriguésia.

[40] Gonçalves CN, Mesquita FW, Lima NRG, Coslope LA, Lintomen BS (2011). Recorrência dos Incêndios e Fitossociologia da Vegetação em Áreas com Diferentes Regimes de Queima no Parque Nacional da Chapada Diamantina. Biodiversidade Bras..

[41] Santos SNA (2011). Conquista e Resistência dos Payayá no Sertão das Jacobinas: Tapuias, Tupi, colonos e missionários (1651-1706).

[42] Martins RO (2013). “Vinha na fé de trabalhar em diamantes.” Escravos e libertos em Lençóis , Chapada Diamantina-BA (1840 – 1888).

[43] Matta PM (2006). O Garimpo na Chapada Diamantina e seus Impactos Ambientais.

[44] Nascimento MM (2008). Do urbano ao rural: um estudo sobre a relação entre “nativos”, os “de fora” e o movimento alternativo no Vale do Capão - Bahia. Soc e Estado..

[45] Abreu DBDO, Santoro FR, Albuquerque UP, Ladio AH, Medeiros PM (2015). Medicinal plant knowledge in a context of cultural pluralism: a case study in Northeastern Brazil. J Ethnopharmacol..

[46] Jacob M (2021). Alimentação e cultura para nutrição.

[47] Canesqui AM (2007). A qualidade dos alimentos: análise de algumas categorias da dietética popular. Rev Nutr..

[48] Albuquerque UP, Soldati GT, Sieber SS, Ramos MA, Sa JC, Souza LC (2011). The use of plants in the medical system of the Fulni-(o)over-cap people (NE Brazil): a perspective on age and gender. J Ethnopharmacol..

[49] Smith JJ, Borgatti SP (1997). Salience counts—and so does accuracy: correcting and updating a measure for free-list-item salience. J Linguist Anthropol..

[50] Ghorbani A, Langenberger G, Sauerborn J. A comparison of the wild food plant use knowledge of ethnic minorities in Naban River Watershed National Nature Reserve, Yunnan, SW China. J Ethnobiol Ethnomed. 2012;8:17. 10.1186/1746-4269-8-17 PMC3485152 22559280

[51] Nascimento VT, Lucena RFP, Maciel MIS, Albuquerque UP (2013). Knowledge and use of wild food plants in areas of dry seasonal forests in Brazil. Ecol Food Nutr..

[52] VT do N, Pereira H de C, Silva AS, Nunes AT, PM de M (2015). Plantas alimentícias espontâneas conhecidas pelos moradores do Vau da Boa Esperança, município de Barreiras, oeste da Bahia, nordeste do Brasil. Ouricuri.

[53] Tabuti JRS, Dhillion SS, Lye KA (2004). The status of wild food plants in Bulamogi County. Uganda. Int J Food Sci Nutr..

[54] RA V, Leony A (2004). Forgetting the forest: assessing medicinal plant erosion in Eastern Brazil. Econ Bot..

[55] Silva NCB, Delfino RAC, Esquibel MA, do ES SJ, Almeida MZ (2012). Medicinal plants use in Barra II quilombola community - Bahia, Brazil. Bol Latinoam y del Caribe Plantas Med y Aromat.

[56] Hora JSL, Feitosa IS, Albuquerque UP, Ramos MA, Medeiros PM (2021). Drivers of species’ use for fuelwood purposes: a case study in the Brazilian semiarid region. J Arid Environ..

[57] Albuquerque UP, de Medeiros PM, Ferreira Júnior WS, da Silva TC, da Silva RRV, Gonçalves-Souza T (2019). Social-ecological theory of maximization: basic concepts and two initial models. Biol Theory.

[58] Hill K, Kaplan H, Hawkes K, Hurtado AM (1987). Foraging decisions among Aché hunter-gatherers: new data and implications for optimal foraging models. Ethol Sociobiol..

[59] von Essen E, Mårtensson F (2017). Young adults’ use of emotional food memories to build resilience. Appetite. Elsevier Ltd.

[60] Hora JSL, Silva TC, Nascimento VT (2020). “É natural, é bom! são frutos que vem da natureza”: representações locais sobre o consumo de plantas alimentícias silvestres em uma área rural do Brasil. Ethnoscientia..

